# Diagnostic performance of additional imaging tests for staging purposes in a bicentric German series of low-risk early breast cancer patients

**DOI:** 10.1007/s00404-023-07169-4

**Published:** 2023-09-07

**Authors:** Lisa Jung, Sarah Isabelle Huwer, Florin-Andrei Taran, Clara Unger, Carolin Müller, Erich-Franz Solomayer, Ingolf Juhasz-Böss, Jakob Neubauer

**Affiliations:** 1 Department of Obstetrics & Gynecology at the Medical Center–University of Freiburg, Hugstetterstr. 55, 79106 Freiburg, Germany; 2 Department of Obstetrics and Gynecology at the Medical Center, University of Saarland, Homburg, Germany; 3 Department of Radiology, University Medical Center Freiburg, Freiburg, Germany

**Keywords:** Breast cancer, Staging, Follow-up, Low risk, Distant metastasis

## Abstract

**Purpose:**

Low-risk early breast cancer rarely leads to the development of metastatic disease, and in these patients, additional imaging test is controversial. The aim of our study was to evaluate the conventional staging procedures in a bicentric German series of low-risk breast carcinoma patients.

**Methods:**

Retrospective evaluation of all patients diagnosed with early, low-risk breast cancer at Saarland University Hospital and Freiburg University Hospital in 2017 was performed. Clinical patient characteristics, the number and type of additional imaging examinations, follow-up examinations, and results were evaluated. The detection rate of metastases and the rate of false-positive findings were analyzed.

**Results:**

A total of 203 patients were included, with all patients received at least one additional imaging test. Initially, a total of 562 additional imaging examinations were performed: 166 chest X-rays, 169 upper abdominal ultrasounds, 199 bone scans, 27 computer tomographies (CT) chest and abdomen, and 1 CT abdomen. 6.8% of patients had abnormal findings reported, requiring 38 additional imaging examinations. One patient (0.5%) was found to have bone metastases. The rate of false-positive findings in the performed additional imaging procedures was 6.6%.

**Conclusion:**

Metastatic disease was detected in one of 203 patients with low-risk early breast cancer. A total of 562 examinations and additional 38 follow-up examinations were performed without detection of metastasis (this corresponds to approximately 3 examinations/patient). The rate of false-positive findings was 6.6%. The performance of additional imaging procedures for detection of distant metastases should be critically reconsidered in patients with low-risk early breast cancer.

## What does this study add to the clinical work

**Table:** 

Staging in newly diagnosed, low-risk breast cancer is not recommended. If additional imaging seems necessary, imaging procedures with high quality are obligate to avoid unnecessary additional examinations for the patients.	

## Introduction

Breast cancer is the most common malignancy worldwide. In 2020, 2.3 million people worldwide were diagnosed with breast cancer, and according to the World Health Organization (WHO), breast cancer incidence will increase in the next years [1]. It is assumed that, by 2040, 3 million people will be newly diagnosed with breast cancer each year [1].

Detection of distant metastases is important for treatment planning and assessment of prognosis [2]. However, the incidence of distant metastases at the initial presentation is low with approximately 4% [3]. Especially, patients with low-risk, early breast cancer (EBC) are unlikely to metastasize early [4]. Regarding cancer subtype, hormone receptor positive, Her2 negative, node-negative breast cancer with low tumor stage has good prognosis [5]. In recent years, multigene prognostic tests have been integrated into everyday clinical practice to further improve treatment and spare additional treatments (e.g., chemotherapy) to patients who do not benefit [6, 7].

Because of the low pretest probability, studies of additional imaging procedures (e.g.) with poor diagnostic accuracy in this clinical setting result in a relatively high rate of false-positive findings [3]. Therefore, the current guidelines do not recommend staging in low-risk breast cancer without clinical suspicion of distant metastases [2].

Breast cancer metastasizes most often lymphogenic (local), less commonly hematogenic, and then predominantly to bone, lung, liver, and brain [8]. Until recently, it was part of the clinical routine at Saarland University Hospital and Freiburg University Hospital to perform additional imaging procedures in every patient with breast cancer. In the absence of clear recommendations in guidelines, the choice of examinations was individual and could include upper abdominal ultrasound, chest X-ray, and bone scans [3]. Rarely, computed tomography of the thorax and abdomen was also performed [3].

Within different health care systems, additional imaging procedures for EBC are performed heterogeneously [3]. Therefore, we wanted to evaluate to what extent staging examinations were performed in patients with low-risk breast carcinoma in two centers in Germany. We were also interested in how effective this conventional staging approach was in terms of the number of metastases detected and the rate of findings clinically considered false positive. In addition, we were interested in the resulting number and type of follow-up examinations.

## Methods

2017 was the last year in which all low-risk breast cancer patients at Saarland University Hospital and Freiburg University Hospital underwent routinely additional imaging procedures to exclude metastatic disease. Therefore, all patients treated with a primary diagnosis of EBC at Saarland University Hospital and Freiburg University Hospital in 2017 were evaluated. Data were obtained from clinical records as well as tumor registries (Department of Obstetrics & Gynecology at the Medical Center—University of Freiburg und Department of Obstetrics & Gynecology of Saarland). Data were analyzed using Microsoft Excel 2010 (Microsoft, Redmond, WA, USA).

**Fig. 1:**
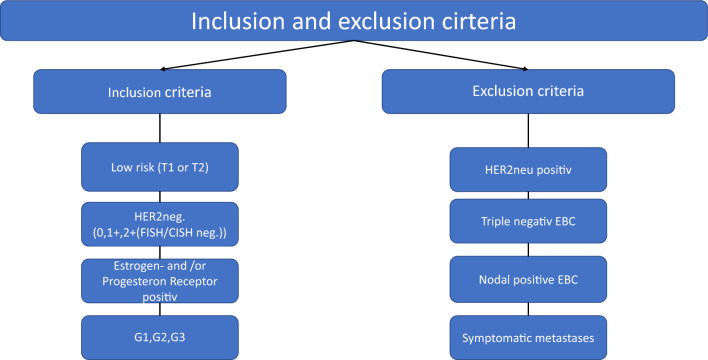
Inclusion and exclusion criteria

All patients with low-risk EBC were included in this retrospective data analysis. Low-risk EBC was defined as follows: T1 or T2 stage, Her2neu negative (0 or 1 + or 2 + with negative FISH/CISH), estrogen and/or progesterone receptor positive, nodal negative (N0), and grading G1, G2, or G3. Patients with triple-negative EBC, Her2neu-positive EBC, nodal-positive EBC, or patients with symptomatic metastases were not included (Fig. 1).

In addition to patient and tumor characteristics, the number and type of additional imaging examinations and the resulting additional examinations were evaluated for all patients. Additional imaging examinations were those that were initially performed after diagnosis of EBC; these included: chest X-ray, upper abdominal ultrasound, and/or bone scan; in some cases, a CT (computed tomography)-thorax/abdomen was also performed initially. Follow-up examinations resulting from conspicuous initial additional procedures included: CT thorax, MRI (magnetic resonance imaging) of the liver, CT of the pelvis or thoracic spine, cranial computed tomography (CCT), radiographic examination of the femur and humerus, bone scan controls, MRI of the thigh, or radiographic examination of the bony hemithorax. Timewise, all examinations between diagnosis and final clinical evaluation of any conspicuous or abnormal findings were considered. The detection rate and rate of false-positive findings of the performed imaging procedures were determined. Written informed consent for data collection for clinical and research purposes was obtained from each participating patient. This complied with local ethical standards.

## Results

The data of 547 consecutive patients who underwent treatment for EBC at Saarland University Hospital and Freiburg University Hospital with an initial diagnosis in 2017 were analyzed. Of these, a total of 203 (37.1%) patients with low-risk EBC could be included in the study. The characteristics of the patients are shown in Table 1. The mean age at initial diagnosis was 61.2 years. Table 2 shows the initial additional procedures performed to exclude metastatic disease.

**Table 1:** Characteristics of the patients

Characteristics of the patients	
Mean age	61.2 years
Stage	
T1	155 (76.4%)
T2	48 (23.6%)
Nodal status	
N0	203 (100%)
Metastatic disease	
M0	202 (99.5%)
M1	1 (0.5%)
Grading	
G1	88 (43.3%)
G2	84 (41.4%)
G3	31 (15.3%)
Estrogene receptor status	
Positive	199 (98.0%)
Negative	4 (2.0%)
Progesterone receptor status	
Positive	183 (90.1%)
Negative	20 (9.9%)
Her2neu receptor status	
Positive	0
Negative	203 (100%)

**Table 2:** Additional imaging procedures at initial diagnosis of EBC*CT = computed tomography

Procedure	Done	*n* (%)
*Additional imaging procedures at initial diagnosis of EBC (n = 203 patients)*		
Chest radiography	Yes	166 (81.8%)
	No	37 (18.2%)
Sonography of the upper abdomen	Yes	169 (83.3%)
	No	34 (16.7%)
Bone scan	Yes	199 (98.0%)
	No	4 (2.0%)
*CT thorax/abdomen	Yes	27 (13.3%)
	No	176 (86.7%)
*CT abdomen	Yes	1 (0.5%)
	No	22 (99.5%)

A total of 166 (81.8%) patients underwent chest radiography, of which abnormal findings were described in 10 (6.0%) patients (Table 2). These were further clarified by means of an additional CT chest examination, all of which were unremarkable (Table 3).

**Table 3:** Follow-up imaging procedures

Initially	Conspicuous examinations	Additional imaging examinations	Conspicuous additional imaging examinations
*Follow-up Imaging Procedures (n = 38 Procedures)*			
*n* = 562 (100%)	*n* = 38 (6.8%)	*n* = 38 (100%)	*n* = 4 (10.5%)
Chest radiography * n* = 166 (81.8%)	*n* = 10 (6.0%)	10 × CT thorax	*n* = 0
Upper abdominal sonography *n* = 169 (83.3%)	*n* = 2 (1.0%)	2 × MRI of the liver	*n* = 0
Skeletal scintigraphy *n* = 199 (98.0%)	*n* = 18 (9.0%)	5 × CT thorax	1 × MRl thigh (= metastases)
		2 × CT thorax-abdomen 2xCT pelvis	3 × CT thorax control in 3 month
		1 × CT thoracic spine	
		1 × CCT	
		3 × X-ray femur	
		1 × X-ray humerus	
		2 × Skeletal scintigraphy	
		1×MRI thigh	
		2 × X-ray bony thorax	
CT thorax-abdomen *n* = 27 (13.3%)	*n* = 7 (25.9%)	1 × MRI liver	*n* = 0
		4 × CT thorax control	
		(in 3 month)	
		1 × liver sonography	
CT abdomen *n* = 1 (0.5%)	*n* = 0	*n* = 0	*n* = 0

Upper abdominal sonography was performed in 169 (80.3%) patients (Table 2). Two patients (1.0%) that underwent sonography of the upper abdomen showed findings requiring clarification, which could not be confirmed by an additional liver MRI (Table 3).

Bone scan was performed in 199 (98.0%) patients (Table 2). A total of 18 (9.0%) patients that underwent bone scan showed conspicuous findings (Table 3). Metastatic disease was ruled out by additional examinations such as a CT thorax (*n* = 5), a chest and abdominal CT (*n* = 2), a pelvis CT (*n* = 2), a CT of the thoracic spine, a CCT, an X-ray examination of the femur and humerus, follow-up skeletal scintigraphies, an MRI of the thigh, and an X-ray examination of the thorax (Table 3). The thigh MRI scan demonstrated a metastasis in the femur (0.5%) (Table 3).

CT thorax detected suspicious findings of the lung in three patients (1.5%), with the recommendation to be monitored by further follow-up 3 months later (Table 3). CT thorax control was performed in all three patients after 3 months. However, no diagnosis could be made in any patient, and a check-up was again recommended in 6 months (Table 3). All other examinations were unremarkable (Table 3).

A CT thoracic abdomen was initially performed in a total of 27 (13.3%) patients (Table 3). Here, seven (25.9%) presented findings requiring clarification. These were clarified by liver MRI, a CT thorax, and a liver ultrasound (Table 3). All of these additional examinations were unremarkable (Table 3).

Table 3 summarizes the follow-up examinations. Initially, 562 staging examinations were performed. Abnormalities were described in 38 (6.8%) examinations, so that an additional examination was recommended for further differentiation (Table 3). Subsequently, 38 additional imaging examinations were ordered. Metastasis was detected in one examination. This means that a total of 562 examinations and additional 38 follow-up examinations were performed without evidence of metastasis. Only one patient was diagnosed with distant metastasis by staging examinations in a conspicuous additional imaging examination. Thus, distant metastasis was detected in one of 203 patients in our collective. This corresponds to a detection rate of 0.5%, meaning that 99.5% of all examinations were carried out without the detection of abnormalities. The rate of false-positive findings was 6.6% (37 out of 562 examinations).

## Discussion

In this retrospective study of 203 patients from a bicentric German patient series with EBC and low-risk profile, we found that the detection rate of distant metastases is very low with additional imaging procedures performed at the initial diagnosis and consisting of chest radiography, abdominal ultrasonography, and bone scan. The study also showed that this form of staging leads to follow-up imaging in a relatively high number of cases (6.8%). On the other hand, the rate of findings clinically considered false positives is very high in this situation (99.5%).

Staging examinations are important for patients with an initial diagnosis of breast cancer to plan therapy and assess prognosis. However, the rate of metastatic disease is low at initial diagnosis of early breast cancer with low-risk profile [9]. Therefore, there was an ongoing discussion regarding the benefit of whole-body staging in low-risk EBC [10, 11]. In the study by Schneider et al. (2003), distant metastasis was detected in tumors smaller than 1 cm in only 3.9% of cases [12].

In various publications, imaging examination methods were presented individually and only a low detection rate for metastases at the time of initial diagnosis was found for bone scan with 0.5–11% [12–14], for liver ultrasound with 0.24–3.3% [12–14], and for chest radiography 0.2–1.2% [12, 13]. In the study by Schneider et al., a few metastases were detected at the pT1 stage; only 2 of 106 (2.7%) patients at the pT1c stage were shown to have a bone metastasis [12]. This is also confirmed in our collective: distant metastases were detected in only one patient, which corresponds to a detection rate of 0.5%.

Based on these data, the current guidelines no longer recommend whole-body staging in patients with early breast cancer [15–17]. However, the extent to which there are exceptional cases in which staging should be performed despite low-risk carcinoma is unclear. Symptomatic patients should be evaluated in any case. The current S3 guideline only recommends staging (in the case of aggressive tumor biology) if these examinations would have a decisive influence on the therapeutic procedure [16]. The current AGO guideline recommends whole-body staging only in cases of high risk for distant metastases and/ or symptoms and/or indication for (neo-)adjuvant chemo/antibody therapy [18].

The lack of studies showing an impact of staging on outcome (survival or progression-free survival) is repeatedly pointed out [16]. However, since staging is a diagnostic test, according to the Centre for Evidence-based Medicine, valid data on the diagnostic accuracy of the test are required, not outcome data. The background for this is that there are multiple confounders (different handling of test results/different therapies) between diagnostic test (staging) and outcome. In addition, each test generates new patient groups for whom adequate therapy may not yet have been evaluated. Therefore, the valid assessment of the quality of a diagnostic test via outcome parameters is not reasonably possible in most cases.

Data on diagnostic accuracy are available for many imaging modalities. However, the procedures mainly used in our study are characterized by a relatively low diagnostic accuracy. When procedures with relatively low diagnostic accuracy are used, a high rate of false-positive findings occurs. This was also observed in our study. The effect was amplified by the low pretest probability of our low-risk collective. Furthermore, if patients undergo staging via sonography of the upper abdomen, bone scan, and chest radiography, it often requires the patients to arrange multiple appointments [3]. Whereas the CT thorax/abdomen is logistically easier for the patients, as they already tend to have multiple doctors’ appointments for therapy planning and start of treatment [3].

To our knowledge, we looked for the first time at the resulting follow-up caused by the conventional staging in low-risk breast carcinomas. Overall, this work described abnormal chest radiographic findings in ten (6%) patients and two (1.0%) upper abdominal sonographies requiring clarification. Eighteen (9%) patients had suspicious findings on skeletal scintigraphy, so further follow-up examinations were recommended. Thus, a total of 599 examinations could be performed without evidence of metastasis. The false-positive rate in our collective was 6.8%, with no difference between the respective examination methods.

These diagnostic measures are a burden for the patients concerned, especially the psychological impact caused by this diagnosis and the resulting consequences [17]. Additional radiation exposure may not be without risk either [19]. However, at the current time, there is no evidence that diagnostic imaging causes malignancies.

Three patients in our collective underwent CT thoracic examinations at 3-month intervals, because the findings did not provide clear evidence of metastasis. Another important issue is also the cost incurred by these diagnostic measures. In the study by Eismann et al. (2013), costs for staging without benefit were calculated at 5–20 million euros/year [20].

Additional resources are wasted by the multitude of unnecessary examinations which could possibly benefit patients for whom these examinations would be more necessary. In addition, it should be mentioned that staging examinations may delay the start of therapy [21].

One possible solution is the use of imaging techniques with better diagnostic accuracy. Because of better sensitivity and specificity, CT thorax/abdomen and a skeletal scintigram have replaced the former staging with X-ray thorax and abdominal ultrasound as basic staging examinations (ESMO 2015/17) [16]. Also debatable would be the use of PET-CT, PET-MRI, or whole-body MRI, which have significantly better diagnostic accuracy compared with the methods used in this study [22].

In this work, only low-risk carcinomas were evaluated, and our collective included all patients with a grading of 1, 2, or 3. Neither the S3 guideline nor the AGO guideline specifically addresses the importance of staging examinations in G3 carcinomas [16, 18]. However, the St. Gallen International Consensus Conference of 2017 already classified G3 tumors as a relative indication for chemotherapy [23, 24], and therefore, pretherapeutic staging is recommended in these patients [25]. However, in special cases, e.g., very small tumors (pT1a), staging and chemotherapy can be dispensed [23, 24].

Not all patients in our collective received a complete staging. In some cases, only one or two metastatic sites were investigated. The indication for the respective examination or the omission of an examination can no longer be precisely traced due to the retrospective data evaluation and must be self-critically evaluated as a weakness of the work.

## Conclusion

Because of the low detection rate (1 in 203), the conventional staging with imaging modalities of low diagnostic quality should not be performed in breast cancer patients with low-risk profiles.
